# Unexpected complexity in the interference activity of a cloned influenza defective interfering RNA

**DOI:** 10.1186/s12985-017-0805-6

**Published:** 2017-07-24

**Authors:** Bo Meng, Kirsten Bentley, Anthony C. Marriott, Paul D. Scott, Nigel J. Dimmock, Andrew J. Easton

**Affiliations:** 1 Present Address: Department of Medicine, Addenbrooke’s Hospital, Hills Road, Cambridge, CB2 0QQ UK; 2 Present Address: Biomedical Sciences Research Complex, North Haugh, University of St. Andrews, St Andrews, KY16 9ST UK; 3 Present Address: Public Health England, Porton Down, Salisbury, SP4 0JG UK; 4 Present Address: Public Health England Birmingham Microbiology, Department of Pathology, Heart of England NHS Foundation Trust, Heartlands Hospital, Bordesley Green East, Salisbury, B9 5SS UK; 5 School of Life Sciences, University of Warwick, Coventry, CV4 7AL UK

**Keywords:** Influenza virus, Defective interfering RNA, Replication, Interference

## Abstract

**Background:**

Defective interfering (DI) viruses are natural antivirals made by nearly all viruses. They have a highly deleted genome (thus being non-infectious) and interfere with the replication of genetically related infectious viruses. We have produced the first potential therapeutic DI virus for the clinic by cloning an influenza A DI RNA (1/244) which was derived naturally from genome segment 1. This is highly effective in vivo, and has unexpectedly broad-spectrum activity with two different modes of action: inhibiting influenza A viruses through RNA interference, and all other (interferon-sensitive) respiratory viruses through stimulating interferon type I.

**Results:**

We have investigated the RNA inhibitory mechanism(s) of DI 1/244 RNA. Ablation of initiation codons does not diminish interference showing that no protein product is required for protection. Further analysis indicated that 1/244 DI RNA interferes by replacing the cognate full-length segment 1 RNA in progeny virions, while interfering with the expression of genome segment 1, its cognate RNA, and genome RNAs 2 and 3, but not genome RNA 6, a representative of the non-polymerase genes.

**Conclusions:**

Our data contradict the dogma that a DI RNA only interferes with expression from its cognate full-length segment. There is reciprocity as cloned segment 2 and 3 DI RNAs inhibited expression of RNAs from a segment 1 target. These data demonstrate an unexpected complexity in the mechanism of interference by this cloned therapeutic DI RNA.

## Background

Viral replication frequently generates progeny genomes that contain extensive deletions [1–4]. This is thought to be a consequence of the polymerase detaching from the initial template and reattaching elsewhere on the same or different template. The truncated genomes are unable to generate infectious virus particles and are thus functionally defective. During a single infectious cycle a large number of different defective genomes can be generated, each lacking a different portion of the genome. Molecules containing the necessary signals will be replicated and packaged into virus particles. Some, but not all, of these defective genomes are also capable of interfering with the growth of the parental virus from which they were derived and are known as defective-interfering (DI) genomes [5]. The ability to interfere with virus multiplication suggests that these DI genomes could act as antiviral agents in the clinic [6, 7].

The influenza A virus genome comprises 8 segments of single-stranded negative-sense RNA (vRNA) in the form of ribonucleoprotein (RNP) complexes, and one copy of each of these segments is required to make an infectious virus particle [8–10]. Influenza virus infections can generate DI RNAs, which can interfere with virus replication. No copyback or snapback influenza DI RNAs have been detected, and most influenza DI RNAs have a major (approximately 80%) internal deletion, while retaining the *cis*-acting signals required for replication and packaging into virus particles [11–13]. DI RNA is incorporated into a particle but the resulting DI virus cannot replicate autonomously since it is unable to synthesise the protein(s) normally encoded by the deleted segment. Replication of a DI virus thus requires complementation by infectious virus. However, DI RNAs may be able to express truncated proteins which could play a role in interference of infectious virus replication [14, 15].

Influenza virus genome replication commences with synthesis of positive-sense (cRNA) copies of the vRNAs of the infecting virus, and in turn these are used as templates for synthesis of new vRNAs [10]. vRNA is also used as the template for mRNA transcription. Unlike cRNA synthesis, mRNA synthesis is initiated using a primer cleaved from the capped 5′ end of host mRNAs and its synthesis terminates before the end of the template vRNA, prior to polyadenylation [16–19]. Thus the mRNA differs from cRNA in having the primer-derived 5′-extension, being truncated, and being polyadenylated at the 3′ end. The non-coding termini of each segment are crucial for RNA synthesis, and contain a conserved, approximately 12 nucleotide (nt), sequence at the 5′ end which is almost exactly complemented at the 3′ end [10].

Synthesis of influenza virus RNAs is carried out by a virus-encoded RNA-dependent RNA polymerase present within a RNP complex consisting of the vRNA or cRNA in association with the virus NP protein and the heterotrimeric virus RNA polymerase comprised of the PB1, PB2 and PA proteins. The latter are encoded by vRNA segments 2, 1 and 3, respectively. Mutations in PB2 and PA can decouple transcription (mRNA synthesis) from replication (vRNA and cRNA synthesis), such that a mutant polymerase is defective in replication but not in transcription or vice versa [20, 21]. Although initiation of mRNA and cRNA synthesis differ in mechanism, a ‘switch’ directing the polymerase to synthesise one or the other has not been clearly demonstrated, and both can be synthesised from vRNP in a cell-free system [22, 23]. The polymerase complex binds to conserved sequences at the 5′ and 3′ ends of vRNA and cRNA [24–26]. The termini of cRNA differ from those of vRNA at several nucleotide positions, and critical residues of each may interact with distinct regions of the PB1 protein [27, 28]. Synthesis of vRNA and cRNA can also be affected differentially by mutations in the NP or NS1 proteins [29–31], or by the presence or absence of the NS2 protein [32].

Little progress has been made towards understanding the mechanism of interference by DI viruses. For snapback/copyback vesicular stomatitis virus (VSV) DI RNAs that have a high degree of double-strandedness, but not VSV DI RNAs generated by central deletion, the key factor in interference seems to be the extent of terminal complementarity of the DI RNA such that a longer DI RNA with more extensive terminal complementarity out-competes a shorter DI RNA of the same structure [33–35]. In addition, lack of a transcriptional promoter in snapback/copyback DI RNAs gives them a replicative advantage over full-length genomic RNA or centrally deleted DI RNAs that can carryout transcription [3, 13, 36–39]. These properties may explain the observed preponderance of rhabdovirus copyback/snapback DI RNAs. For DI RNAs generated by a central deletion, as seen in influenza viruses, it has been suggested that interference with RNA synthesis involves competition between the DI RNA and genomic RNA for limiting viral or host factor(s), and/or the much shorter DI RNA may have a more rapid rate of synthesis than its cognate genomic RNA giving it a competitive advantage, although there is little experimental evidence supporting this [3, 13, 36–40].

Most studies of DI influenza virus-mediated interference to date have been carried out with naturally occurring preparations, and are compromised by the presence of mixtures of several different defective RNA sequences [4, 41]. This problem was solved using reverse genetics to generate virus stocks containing a molecularly defined naturally occurring DI RNA. Called 1/244 to denote its derivation from segment 1 and the number of nucleotides retained at the 3′ end of the positive-sense RNA, it is propagated and efficiently maintained by infectious virus often referred to as ‘helper’ virus. When delivered intranasally as an influenza virus particle, 1/244 DI virus confers complete protection in mice from a lethal challenge with several different subtypes of influenza A virus (homologous protection) and is more effective than oseltamivir in ferrets in combatting pandemic influenza A/California/04/09 [42]. However, the molecular basis of this homologous protection by 1/244 DI virus is not known. In addition, 1/244 DI virus also protects from the genetically heterologous influenza B virus and a murine pneumovirus in a dose-dependent manner through its ability to induce the local expression of interferon type I, although induction of interferon is not required for protection against influenza A virus [43, 44]. Here we show that interference with influenza A virus mediated by molecularly cloned 1/244 DI RNA does not require expression of a protein product from the deleted segment 1 but results from both competition with the packaging of full-length cognate virion segment 1 RNA, and by inhibiting the synthesis and/or accumulation of RNA directed by segment 1 RNA. Unexpectedly 1/244 DI RNA also interfered with the expression of RNAs from full-length segments 2 and 3, which encode the other polymerase proteins, but not from full-length segment 6.

## Methods

### Plasmids

**Fig. 1:**
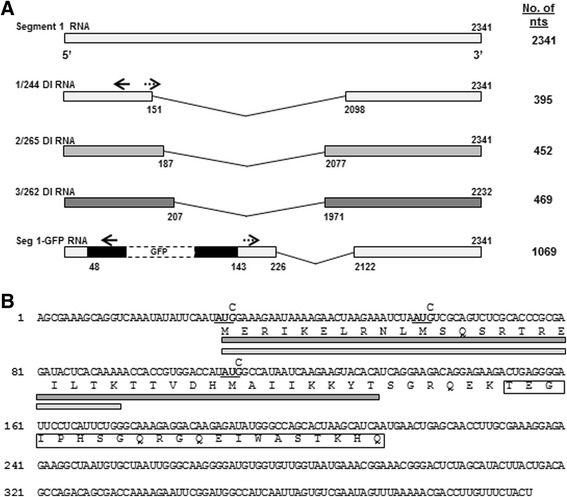
**a.** Schematic diagram of influenza segment 1244 DI RNA (1/244), segment 2265 DI RNA (2/265), segment 3262 DI RNA (3/262), and the Seg 1-GFP RNA expressed from plasmids. The names derive from the 244, 265 and 262 nt remaining at the 3′ end of the cognate vRNAs respectively.The total number of nucleotides comprising each RNA is shown on the right. The nucleotide positions of the breakpoints that result in the deleted genome RNAs used in this study (positive-sense, 5′ to 3′) are indicated. Solid arrows indicate the primers used in the primer extension assays for cRNA and mRNA analyses and dashed arrows indicate primers used for vRNA analyses. For the construction of Seg 1-GFP RNA see Methods. **b.** Sequence of 1/244 DI RNA in cRNA sense indicating the open reading frame, and the predicted protein sequence in single letter amino acid code. The 35 residue PB1 binding domain of PB2 is indicated by the dark grey box, and the 22 residue mitochondrial interaction domain of PB2 is indicated by the light grey box. The boxed amino acid sequence downstream from the central deletion that gave rise to 1/244 DI RNA do not arise from the PB2 ORF. The three G → C mutations at nucleotide positions 30, 60 and 111 used to mutate the in-frame AUG initiation codons are shown in bold and underlined

The plasmids encoding the 8 gene segments of the A/WSN strain of A/WS/33 (H1N1) and plasmids expressing the polymerase proteins and NP [45], and the construct expressing 1/244 DI RNA (1/244), derived from a naturally occurring DI virus, were as previously described [46]. 1/244 RNA comprises 395 nt with 151 nt from the 5′ end and 244 nt from the 3′ end of the positive-sense cognate RNA and was derived naturally from segment 1 of A/Puerto Rico/8/34 (H1N1) (Fig. 1). The segment 1 target, segment 1-GFP, expressing the green fluorescent protein (GFP), was created by amplifying the GFP open reading frame by PCR from pEGFP-N1 (Clontech) using primers 5’ATGGTCTCTACTGATGGTGAGCAAGGGCGAG and 5’ATGAAGACAATCTCTTACTTGTACAGCTCGTCCA. The product was inserted between the *BpiI* and *Eco*31*I* sites of pPolI-220 [47] such that the GFP ORF is in-frame with the PB2 ORF, giving plasmid seg 1-GFP which expresses segment 1-GFP RNA (Fig. 1). The GFP reporter retains the exact 5′ (220 nt) and 3′ (48 nt) terminus of segment 1 and is cognate for 1/244 DI RNA. A naturally occurring segment 2 DI (2/265; comprising 452 nt in total with 265 nt from the 3′ end and 187 nt from the 5′ end of the positive-sense cognate RNA) was isolated from a DI A/equine/Newmarket/7339/79 (H3N8) virus preparation (Fig. 1) [48] by RT-PCR amplification, and subsequently cloned into a pPolI-SapIT expression vector [49]. A further naturally occurring segment 3 DI RNA (3/262; comprising 469 nt in total with 262 nt from the 3′ end and 207 nt from 5′ end of the positive-sense cognate RNA) was isolated from a DI A/WSN preparation, and was amplified and cloned as above (Fig. 1). The DI RNAs encoded by the various plasmids retain the exact nucleotide sequences from the termini of the genome segments of the viruses from which they were derived and do not contain any mutations in positions known to have an effect on replication or packaging. Two sequential steps of site-directed mutagenesis were carried out to mutate the start codons in the 1/244 DI RNA. A pair of primers (GTTCTTTTATTCTTTCgATATTGAATATAATTG and CAATTATATTCAATATcGAAAGAATAAAAGAAC) was used for site-directed mutagenesis to convert the first AUG to AUC using a pPolI-244 plasmid as template and Pfu DNA polymerase (Promega). The lower case letters indicate the position of the mutations. The second round of site-directed mutagenesis used primers that altered the second and third start codons to AUC using the construct produced from the first round of mutagenesis (CTTCTTGATTATGGCcATATGGTCCACGGTGGTTTTTGTGAGTATCTCGCGGGTGCGAGACTGCGAcATTAGATTTCTTAGT and ACTAAGAAATCTAATgTCGCAGTCTCGCACCCGCGAGATACTCACAAAAACCACCGTGGACCATATgGCCATAATCAAGAAG). All constructs were confirmed by sequencing.

### Transfection and recovery of infectious virus

Human 293T cells (American Type Culture Collection) were transfected as previously described. Briefly, for northern blot analysis, a well of 70% confluent 293T cells in a 12-well plate was transfected using TransIT LT1 transfection reagent (Mirus) with 8 PolI expression plasmids encoding virion sense RNA, and cDNA plasmids for expression of PB2, PB1, PA and NP proteins, with or without pPolI-244. The transfected cells were then incubated at 37 °C overnight before co-culture with MDCK cells (obtained from the American Type Culture Collection) in a 25 cm^2^ flask. Total cellular RNA was extracted with 2 ml Trizol reagent per sample (Invitrogen) on days 1, 2 and 3 after co-culture while tissue culture fluid was collected for virus titration and RNA extraction. Virions were purified by ultracentrifugation. RNA was extracted with phenol/chloroform, and then ethanol precipitated.

For preparation of virus stocks containing DI 1/244 and 1/244 KO DI RNA, virus in tissue culture fluids produced by transfection was passaged once in embryonated chicken’s eggs and allantoic fluids were harvested to produce a stock of virus. The virus produced in embryonated chicken’s eggs is a mixture of 1/244 DI virus or 1/244 AUG KO DI virus packaged in A/WSN virion proteins and infectious helper A/WSN strain of A/WS/33 (H1N1) virus. These were purified by differential centrifugation through sucrose, and resuspended in PBS. Stocks were standardized according to their haemagglutination titre and stored in liquid nitrogen. The DI virus stock was UV-irradiated to remove helper virus infectivity using a short burst (40 s) of UV irradiation at 253.7 nm (0.64 mW/cm^2^). This is ‘active DI virus’. Longer UV irradiation (8 min) inactivates the protective activity in mice, but does not affect haemagglutinin or neuraminidase activities, and so controls for any immune system-stimulating or receptor-blocking effects of 1/244 DI virus particles (‘inactivated DI virus’).

### Infectivity assay

MDCK cell monolayers in 96-well plates were infected with supernatant containing rescued A/WSN as described previously [44]. After 1 h for attachment of virus, the monolayer was washed with PBS, and incubated in maintenance medium overnight at 33 °C. Cells were then fixed with 4% (*v*/v) formaldehyde, washed and blocked with 5% (*w*/*v*) milk powder in PBS. The infected cells were probed with a monoclonal antibody specific for the HA of A/WSN in PBS containing 0.1% Tween 20. After washing, goat anti-mouse IgG–alkaline phosphatase conjugate (Sigma) in TBS containing 0.1% Tween 20 was added, and infected cells were detected with nitrotetrazolium blue chloride/BCIP (Sigma) in Tris-buffered magnesium chloride and sodium chloride (0.1 M, pH 9.5). The infectivity titre was determined by counting at least 50 positively stained cells (foci) at an appropriate dilution in each of the triplicate wells. The mean number of counts was determined to give a titre in focus-forming units (f.f.u.) ml^−1^.

### Protection of mice from influenza with DI virus

To assess the degree of in vivo protection afforded by DI virus, groups of 5 C3H/He-mg mice were each inoculated intranasally under light ether anaesthesia with A/WSN alone (10 LD_50_ or 1000 f.f.u.), active DI virus alone, a mixture of A/WSN + active DI virus, or A/WSN + inactivated DI virus. The amounts of DI 1/244 used was the same as used in previous studies of protection from disease and mice were subsequently monitored for clinical disease according to our standard protocol [43, 44]. The severity of disease was scored using the standard scoring system with a score of 1 indicating a healthy animal and a score of 5 indicating death with a range of severity of cinical signs for intervening scores as described previously [43, 44]. Animals were assessed daily and data are expressed as the mean score for each group. Surviving mice were challenged 3 weeks after infection with a high dose of A/WSN (10,000 LD_50_) to determine their immune status. All work with animals was governed by a licence approved by the UK Home Office and complied with national regulations. Animal work was approved by the University of Warwick Animal Welfare and Ethical Review Board as required by the Animals (Scientific Procedures) Act (1986) governing animal experimentation in the UK. Animals culled for humane reasons were subjected to an approved procedure to minimise suffering.

### RNA extraction and primer extension

For primer extensions, each well of a 6 well plate containing 70% confluent 293T cells was transfected with 1 μg each of the PB2, PB1, PA and NP cDNA expression plasmids plus various amounts of either a DI plasmid, pPolI-PB2, or pPolI-NA together with 1 μg of target plasmid. Cells were incubated at 37 °C and total cellular RNA was extracted from cells with Trizol at 48 h post-transfection.

Primer extension analysis was carried out as previously described [50], using primer mixes in order to detect vRNA, cRNA, mRNA and 5S rRNA simultaneously from individual samples. Total RNA (2 μg) was mixed with [^32^P]5′-end labelled primers and dNTP in a total volume of 13 μl. The mixture was heated at 65 °C for 5 min and placed on ice for 1 min. 2X first Strand Buffer, 20 mM DTT, and 100 U SuperScript III reverse transcriptase (Invitrogen) were added and further incubated at 55 °C for 1 h. The reaction was terminated by heating at 95 °C for 5 min with gel loading dye II (Ambion). The transcription products were resolved on a 6% (*w*/*v*) polyacrylamide gel containing 7 M urea in TBE buffer and detected by phosphor imaging. The primers used, and the expected product sizes, are shown in Table 1 and the positions of these on the various target RNAs are demonstrated in Fig. 1a.

**Table 1:** Sequences of the primers used in this study. The primers containing mixed nucleotides were designed for detection of both A/PR8 and A/WSN-derived RNAs. Influenza virus mRNAs utilise primers that are 10–13 nt in length and the predicted sizes of the mRNA fragments reflect this heterogeneity

Target RNA	Primer specificity	Primer sequence	Expected product Size/s (nt)
Seg 1-GFP	c/mRNA	GGACACGCTGAACTTGTGG	142/152–5
	vRNA	AGATAAGAGGATAATGGAAATG	242
DI 1/244	c/mRNA	ATATGGTCCACKGTGGTTTTTG	110/120–3
	vRNA	GGAGAAGACTGAGGGGATTC	252
Segment 2 (PB1)	c/mRNA*	TCCATGGTGTATCCTGTTCC	146/156–9
	vRNA*	TGATTTCGAATCTGGAAGGA	134
Segment 3 (PA)	c/mRNA*	TGAGTGCATATTGCTGCAAAT	146/156–9
	vRNA*	TTCTTATCGTTCAGGCTCTT	213
Segment 6 (NA)	c/mRNA*	TCCAGTATGGTTTTGAYTTCCR	160/170–3
	vRNA*	TGGACTAGTGSGAGCATSAT	130
5S rRNA*		TCCCAGGCGGTCTCCCATCC	100

* indicates primers taken from [50]

### Northern blotting

Ten μg of total cellular RNA or 50% of the yield of purified virion RNA from each sample was analysed by glyoxal-agarose gel electrophoresis. Poly A-containing mRNA was selected using a GenElute Direct mRNA preparation kit (Sigma) according to the manufacturer’s instructions. Non-polyadenylated RNA that did not bind to the column during mRNA preparation, and therefore contained the viral cRNA, was retained for further study. The RNA was transferred onto Hybond-N membrane (GE Healthcare) overnight using 20× SSC. The membrane was then baked at 80 °C for 2 h and hybridized with digoxigenin (DIG)-labelled probes overnight. The full-length positive-sense DIG-labelled segment 1, segment 2 and segment 7 probes were transcribed in vitro in the presence of DIG-UTP (Roche) from PCR products containing a T7 promoter. The Roche system with a digoxigenin-specific FAb antibody fragment conjugated to alkaline phosphatase and the chemiluminescent CSPD substrate was used for detection. Blots were exposed to Fuji X-ray film until the desired density was achieved and bands were quantified by densitometry using ImageJ (NIH).

### Quantitation of GFP-expressing cells

Human 293T cells were transfected with the segment 1-GFP RNA expressing plasmid, plasmids expressing PB1, PB2, PA and NP proteins, and increasing amounts of an additional PolI plasmid expressing a DI RNA (1/244, 2/265 and 3/262) or a full-length RNA (segment 1, 4 or 6). At 2 days post-transfection, the cultures were examined for GFP expression. Each dataset was replicated several times and all data were included in the calculations. Digital images of the cell monolayers were taken by phase-contrast and epifluorescence microscopy. Five field fluorescence images were randomly selected and analysed for the proportion of the visualised area expressing GFP using the HCImage software (Hamamatsu). The visualisation detects cells expressing a range of GFP levels to include those that may have been transfected with different levels of the reporter plasmids. A mean was calculated to give the percentage of the GFP positive area per monolayer.

## Results

### 1/244 DI RNA directs synthesis of a small mRNA in the presence of infectious helper virus

**Fig. 2:**
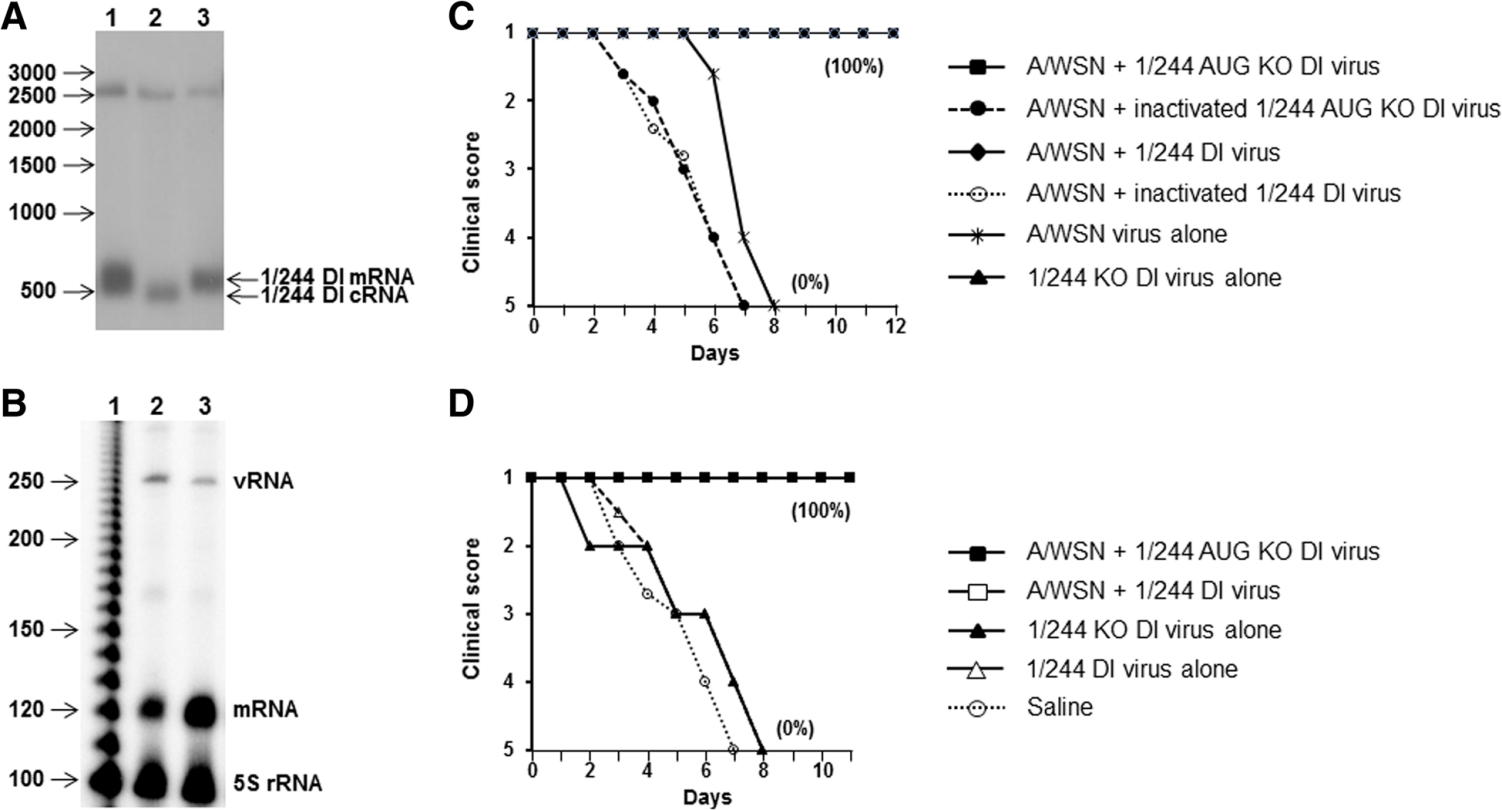
Detection of RNAs synthesised by 1/244 DI RNA and 1/244 KO DI RNA by Northern blot and primer extension and protection of mice from influenza. **a.** Northern blot of RNA extracted from cells 48 h after infection with 1/244 DI virus and helper virus to detect positive-sense influenza RNA transcribed from genome segment 1 and the 1/244 RNA itself. Lane 1: total RNA; lane 2: non-polyadenylated RNA; lane 3 polyadenylated mRNA. The positions of size markers (nt) are indicated. **b.** Viral RNAs synthesised by 1/244 DI RNA and 1/244 AUG KO DI RNA and detected by primer extension. Samples were taken 48 h after transfection with plasmids expressing Seg 1-GFP, PB1, PB2, PA and NP proteins and 1/244 and 1/244 AUG KO DI RNAs. Lane 1: a 10 nt size ladder; lane 2: RNA made in the presence of 1/244 DI RNA; lane 3: RNA made in the presence of 1/244 AUG KO DI RNA. The positions of vRNA and mRNA are indicated. 5S ribosomal RNA was measured as a loading control. **c.** Mice were inoculated intranasally with A/WSN alone (10 LD_50_, 1000 f.f.u.), A/WSN + 1/244 AUG KO DI virus, A/WSN + 1/244 DI virus, A/WSN + inactivated 1/244 AUG KO DI virus, A/WSN + inactivated 1/244 DI virus, or saline alone. Each DI virus comprised 1 μg protein. **d.** Surviving mice were challenged with a high dose of A/WSN (10,000 LD_50_) at 3 weeks post-infection to determine their immune status. Panels C and D show the mean clinical score. In panel C A/WSN + 1/244 DI, 1/244 KO DI only, 1/244 DI only, and mock are all hidden under A/WSN + 1/244 AUG KO DI with a clinical score of 1. The percentage of surviving mice is shown in parenthesis

It has been shown that a truncated form of the flu A PB2 protein is capable of inducing type I interferon and an antiviral state [15]. DI 1/244 RNA contains the 3′ terminal 244 nt of the segment 1 vRNA that may direct mRNA synthesis and would consequently have the potential to encode a truncated protein containing the amino terminus of PB2 (Fig. 1a, b). We therefore investigated if the synthesis of the potential truncated protein from 1/244 DI RNA had a role in interference or protection against influenza in vivo. First, we determined if 1/244 DI RNA could direct the synthesis of mRNA by analysing the viral RNAs present in cells infected with 1/244 DI virus and infectious helper virus. As with all DI viruses 1/244 DI virus is replication incompetent, in this case due to the lack of a functional genome segment 1. To study the viral RNAs synthesised from 1/244 we therefore co-infected cells with 1/244 virus and infectious A/WSN (H1/N1) as helper virus to provide the segment 1 encoded PB2 protein needed to generate 1/244 RNAs. Both viruses were generated entirely from molecular clones by reverse genetics as described previously [51] to avoid the presence of other DI RNAs. Northern blot analysis using a segment 1 specific probe detected two polyadenylated virus mRNAs, of approximately 2.3 and 0.5 kb, in infected cells (Fig. 2a). These sizes are consistent with mRNAs predicted to be derived from the full-length genome segment 1 provided by the helper virus and by 1/244 DI RNA, respectively. The positive-sense RNA seen in the non-polyadenylated RNA fraction is cRNA and, as expected, was slightly smaller than the 1/244 DI-derived mRNA. These data indicate that the 1/244 DI RNA is replicated by the infectious helper virus and acts as a template for both replication and transcription from a fully functional RNP complex equivalent to those found with the genomic RNA segments [23].

### Expression of a putative protein by 1/244 DI RNA is not required for interference in vitro or protection against disease in vivo

The mRNA transcribed from 1/244 DI RNA contains the translation start codon of the PB2 open reading frame 1 (ORF-1). This has the potential to encode a protein comprising the first 41 amino acid residues of PB2 fused to 21 amino acid residues translated from a different reading frame generated as a result of the deletion, making a protein of 62 residues in total (Fig. 1b). This putative protein contains the entire PB1-binding domain of residues 1–35 [33] and the mitochondrial localisation domain of residues 1–22 [52]. We investigated whether the putative protein was required for the previously reported protection from infection by 1/244 DI RNA by mutating the sequence to remove the translation initiation codons. The PB2 ORF has three possible AUG start codons and to be sure there could be no translation initiation all three possible start codons were mutated (to AUC). The new RNA is referred to as 1/244 AUG KO DI RNA.

RNAs were harvested 48 h after transfection of 293T cells with plasmid encoding either the 1/244 DI RNA or the 1/244 AUG KO DI RNA together with plasmids expressing the PB2, PB1, PA and NP proteins. Primer extension analysis, using a mixture of primers to detect both positive- and negative-sense RNAs simultaneously (Table 1), showed that 1/244 and 1/244 AUG KO synthesised both types of RNA with the predicted sizes, confirming that both genomes acted as templates for RNA synthesis in transfected cells. Densitometric analysis followed by normalisation against the 5S rRNA loading control showed that the levels of mRNA synthesized by 1/244 and 1/244 AUG KO DI RNAs were similar (mRNA/5S ratios of 0.87 and 0.9, respectively) confirming that transcription was unaffected by the mutations in the 1/244 AUG KO DI RNA (Fig. 2b).

**Fig. 3:**
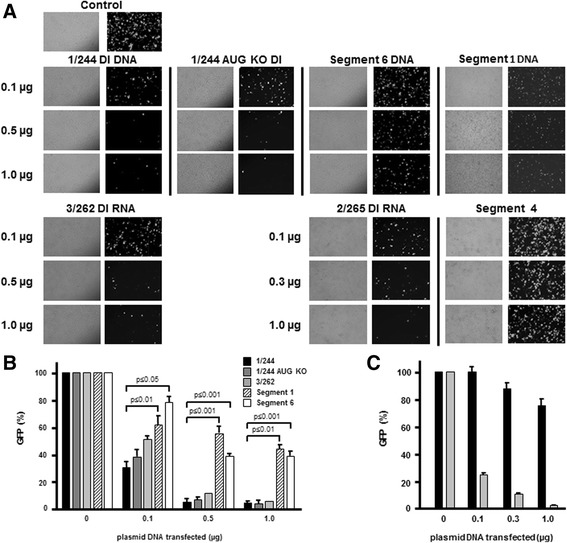
Effect of increasing amounts of DI and full-length genome segment RNAs on expression of GFP from a segment 1 reporter gene construct. 293T cells were transfected with the segment 1-GFP plasmid, plasmids expressing PB1, PB2, PA and NP proteins, and increasing amounts of an additional PolI plasmid expressing a DI RNA (1/244, 1/244 KO, 2/265 or 3/262) or a full-length vRNA (segment 1, 4 or 6). Cells were examined for fluorescence 2 days after transfection. **a.** Pairs of cell monolayer images taken by phase-contrast (left) and epifluorescence microscopy (right). The amount of each plasmid expressing the various RNAs used as putative inhibitors is shown on the left. Control cells (top) were transfected with an empty vector (1 μg). **b.** Quantitation of fluorescence in cells generated in the presence of transfected plasmids expressing 1/244 DI, 1/244 KO DI, 3/262 DI, full-length segment 1 and full-length segment 6 RNAs, as indicated in the key. Columns show the mean of 3 (1/244 RNA, segment 1 and segment 6) or 2 (1/244 KO DI RNA and 3/262) independent experiments, and bars are standard errors of the mean. **c** Quantitation of fluorescence in cells generated in the presence of transfected plasmids expressing full-length segment 4 RNA (black columns) and DI 2/265 RNA (grey columns). Columns show the mean of 2 independent experiments, and bars are standard errors of the mean. Statistical significance determined using a two-tailed Student’s *t* test; *p* values for specific comparisons are shown

In our experience all stocks of influenza virus contain some level of a complex population of DI viruses, which makes examination of the activity of any individual transfected DI RNA in a cell culture infection model technically impossible. In such experiments it is necessary to use a high level of infectious virus which results in a prominent signal from the naturally occurring DI RNAs that obscures the signal from the transfected plasmid expressing 1/244 DI RNA (unpublished data). We have therefore used an established in vitro RNP reconstitution approach [53–55] to examine the ability of DI RNAs to interfere with the expression of green fluorescent protein (GFP) from a Seg 1-GFP construct. Here the coding region of GFP is inserted in-frame into a deleted influenza segment 1 RNA (Fig. 1a). This system also distinguishes effects of DI RNA on RNA synthesis from any effect on RNA packaging as plasmids encoding key structural proteins (HA, NA, M1 and M2) are not included [56]. The interfering ability of 1/244 and 1/244 AUG KO RNAs were determined by transfecting 293T cells with the relevant plasmids and plasmids expressing Seg 1-GFP and PB1, PB2, PA and NP proteins. 1/244 and 1/244 AUG KO RNAs both strongly inhibited fluorescence in a dose-dependent manner (Fig. 3a) with over 90% inhibition at the dose of 0.5 μg for both 1/244 or 1/244 AUG KO plasmids (Fig. 3b).

To determine whether the in vitro interference is also reflected in an infection model, we compared the abilities of 1/244 DI and 1/244 AUG KO DI RNA to protect mice from disease that normally follows challenge with influenza A/WSN (H1N1). Virus stocks containing either 1/244 DI or 1/244 AUG KO DI RNA were produced using reverse genetics as before [51] and mice were infected intranasally with either A/WSN alone, A/WSN and 1/244 DI virus, or A/WSN and 1/244 AUG KO DI virus. Additional infected groups received DI virus which had been UV-irradiated for 8 min to destroy DI protecting activity and to control for any non-specific effects of the DI virus inoculum [43, 44]. Mice were monitored for clinical disease. Figure 2c shows that the A/WSN virus-infected control mice all became seriously ill, and had to be culled. In contrast, none of the infected mice also receiving 1/244 DI virus or 1/244 AUG KO DI virus developed any sign of clinical disease. All mice treated with A/WSN + 1/244 DI virus or 1/244 KO DI virus alone were also healthy throughout and these data points are obscured by those of the A/WSN + 1/244 KO DI virus. As expected from earlier data mice treated with UV-inactivated DI virus were not protected [43, 44]. We have shown previously that animals treated simultaneously with 1/244 DI virus and infectious virus generate protective immunity that prevents disease following subsequent challenge with a high dose of the same virus in the absence of further treatment with DI virus. Animals treated here with 1/244 AUG KO DI virus were solidly immune to further challenge with a high dose of A/WSN showing that they had been infected during the first virus challenge even though they developed no sign of clinical disease (Fig. 2d). These data show that interference in vitro or protection in vivo by 1/244 DI virus does not require expression of a protein product and is a purely RNA-mediated phenomenon.

### 1/244 DI RNA interferes with packaging of segment 1

**Fig. 4:**
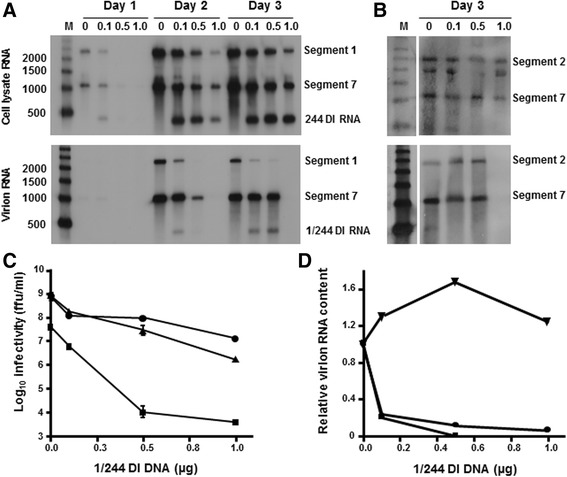
Effect of DI RNA on the accumulation and encapsidation of viral RNAs. 293T cells were transfected with increasing amounts of the 1/244 DI PolI plasmid (0, 0.1, 0.5 and 1.0 μg) and a constant amount of the plasmids needed for the expression of infectious A/WSN virus. After co-cultivation with MDCK cells RNA was extracted from cells and from virus particles purified from culture fluids. **a.** RNA extracted from cell lysates (top panel) and virus particles from supernatants (lower panel) at 1, 2 and 3 days post co-cultivation was analysed with probes specific for segment 1 RNA, segment 7 RNA, and 1/244 DI RNA. The sizes of RNA markers are shown on the left and the identity of the RNAs on the right. **b.** Cell lysate RNA and virion RNA extracted on day 3 were analysed with probes specific for segment 2 RNA and segment 7 RNA. **c.** A/WSN infectivity in cell supernatants measured by microplaque assay. The infectivities on 1 (■), 2 (▲) and 3 (●) days after co-cultivation are shown. Data are the mean of 2 independent experiments with the bar representing the range. **d.** The ratios of levels of segment 1 RNA to segment 7 in virions on days 2 (■) and 3 (●) and of segment 2 RNA to segment 7 in virions on day 3 (▼) were determined by densitometry. These values were compared with the equivalent ratios determined from transfected cells. The ratios of segment 1 or segment 2 in virions over total cellular RNA were calculated and normalised to the value determined in the absence of 1/244 DI RNA

It was reported previously that the interference mediated by a DI virus occured at the level of packaging [57], and we investigated whether this was also the case for DI 1/244. Human 293T cells were transfected with various amounts of the 1/244 DI plasmid and a constant amount of the plasmids needed for the production of infectious A/WSN virus. These cells were then co-cultivated with MDCK cells, and at days 1–3 post co-culture the levels of segments 1, 2 and 7 vRNAs in infected cells and in purified virus particles were determined. Previously we showed that influenza vRNAs were only detectable when all of the virus RNA polymerase components were present, demonstrating that the vRNAs are generated by the virus polymerase [46]. 1/244 DI RNA was observed only in cultures transfected with the 1/244 plasmid, confirming that no other segment 1 DI RNA sequences were generated following transfection (Fig. 4a). We determined the effect of 1/244 DI RNA on the packaging efficiency of full-length segment 1 by measuring the ratio of segment 1 to segment 7 in cells and in virions over time in the absence, or presence of increasing levels, of 1/244 DI RNA. Packaging of segment 7 in virions is expected to be unaffected by a segment 1-derived RNA. Therefore, an alteration in the ratio of intracellular versus packaged RNA segments indicated whether full-length segment 1 was affected by the presence of the DI RNA. Measuring the ratio of segment 1 to segment 7 also controls for any overall reduction in production of infectious virus particles due to the presence of the DI RNA. As the amount of transfected 1/244 DI plasmid DNA was increased there was a progressive reduction of segment 1 in cells (Fig. 4a upper panel) and a corresponding reduction in virus infectivity on each of the days examined (Fig. 4c). This confirmed that 1/244 DI RNA-mediated interference was taking place. The reduction in infectious particle production was mirrored by a reduction in virion RNA levels, with no segment 1, 2 or 7 vRNAs detected in the presence of 1 μg of 1/244 DI plasmid DNA (Fig 4a, b, lower panels). Quantitation showed that in the presence of 1/244 DI RNA, the ratio of full-length segment 1 vRNA: segment 7 vRNA was considerably lower in virions than in cell extracts (Fig. 4d). This established that 1/244 DI RNA acts, at least in part, by selectively excluding full-length cognate segment 1 vRNA from progeny virus particles. The segment 2 vRNA content of virions was not reduced in the presence of increasing amounts of 1/244 plasmid (Fig. 4b, d), confirming that inhibition of packaging of segment 1 by 1/244 DI RNA was specific, and did not extend to other polymerase component-encoding RNA segments. The origin of the band in the total cellular RNA underneath the segment 2 signal is not known, but is likely arise from a non-specific interaction of the probe with cellular RNA.

### Segment 1, 2 or 3 DI RNAs inhibit gene expression from segment 1

To distinguish between the effects of DI RNA on viral RNA synthesis and RNA packaging, GFP expression from the Seg 1-GFP construct was determined as described above. Human 293T cells were transfected with Seg 1-GFP and plasmids expressing PB1, PB2, PA and NP proteins, and GFP fluorescence determined 2 days post-transfection. In addition, increasing amounts of a PolI plasmid expressing either DI 1/244 or an alternative DI RNA (2/265 or 3/262, containing 244, 265 and 262 nt from the 3′ end of the cognate vRNAs, respectively: Fig 1a) or a full-length vRNA not expressing a polymerase component (segment 4 or 6) were transfected. In control experiments, increasing amounts of a PolI plasmid encoding full-length vRNA for segment 1 were added to determine whether the presence of segment 1 sequences in general inhibited GFP expression. In the absence of the expression plasmid encoding the PB2 protein no GFP expression was detected confirming the requirement for formation of a functional vRNP complex (data not shown). Figure 3a shows substantial expression of fluorescence in the absence of any DI RNA, while increasing levels of 1/244 DI led to significantly diminished GFP expression. In contrast, inhibition was far less marked when cells were transfected with plasmids that synthesised full-length segments 1, 4 or 6 vRNAs. The inhibitory effect of 1/244 DI RNA was highly significantly different to that of segment 6 RNA, with 0.1 μg of 1/244 DI plasmid required to inhibit GFP fluorescence by 70%, whereas 10-fold more of the segment 6 plasmid was needed to produce a similar level of inhibition (61–69%) (Fig. 3b). The presence of 1 μg of segment 4 plasmid DNA reduced segment 1-derived gene expression by only 25% (Fig. 3c). Thus 1/244 DI RNA strongly inhibits gene expression from a segment 1 target RNA. Addition of full-length segment 1 had only a relatively small effect with 1 μg plasmid DNA reducing GFP fluorescence by 57%, demonstrating that the effect of the DI plasmids was due to their unique sequences and not to a general effect of segment 1 terminal sequences. Further assays showed that the segment 2- and segment 3-derived DI RNAs (2/265 and 3/262, respectively) also strongly inhibited GFP fluorescence from the segment 1-derived target (Fig. 3a-c), with 1 μg of the 2/265 and 3/262 DI plasmid DNAs reducing expression to 2% and 6% of the control, respectively.

### 1/244 DI RNA inhibits positive-sense RNA synthesised from genome segments 1, 2 and 3 but from not segment 6

**Fig. 5:**
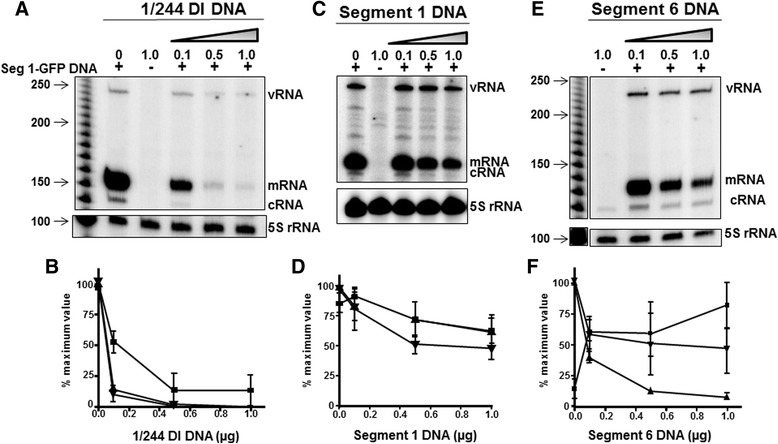
Analysis of influenza segment 1-directed RNA synthesis by primer extension in the presence of influenza 1/244 DI RNA, segment1 RNA, or segment 6 RNA. 293T cells were transfected with plasmids as described in Fig. 3. Primer extension analysis of viral RNA directed by segment 1-GFP in the absence or presence of increasing amounts of plasmid encoding 1/244 DI RNA (panel **a**), genome segment 1 vRNA (**c**) or genome segment 6 (**e**). 5S rRNA detected from the same RNA preparations were used as an internal control. The primer extension products are identified on the left of each panel. Quantitation of viral RNA from three independent experiments by phosphorimaging analysis is shown in panels (**b**), (**d**), and (**f**). The values of band intensities were normalised against the relevant 5S rRNA and are expressed as a percentage of the maximum value for each RNA analysed. Basal levels of vRNA generated from the target plasmid were subtracted from the total as decribed in the text. The error bars represent the standard error of the mean of at least 3 replicates. vRNA (■), mRNA (▲) and cRNA (▼)

Expression of GFP from the Seg 1-GFP PolI plasmid is dependent on transcription of the negative-sense vRNA into mRNA. However, vRNA is also template for cRNA, which in turn acts as template for the production of more vRNA. Influenza virus mRNA has a 5′-extension of approximately 12 nt cleaved from host mRNA [10] and is polyadenylatedso the mRNA and cRNA products are distinguishable by size. Using Seg 1-GFP plasmid with the transfection protocol described above, and a primer extension assay that detects vRNA, mRNA and cRNA, we investigated 1/244 DI RNA mediated interference of RNA synthesis (Fig. 5). Viral RNAs were all of the predicted sizes indicating that the templates generated the correct products. RNA levels were normalised to the 5S loading control levels before quantitation and the levels of vRNA were also adjusted to account for basal transcription from the target plasmids. Basal transcription was calculated as a percentage of the vRNA:5S RNA ratio in the absence of both the plasmid pcDNA-PB2 (which encodes a critical component of the virus polymerase), and of any interfering RNA (see Fig. 7e, lane 1). This value (0.93%) was subtracted from the vRNA levels. Values of zero indicate that no vRNA was detected above the basal level.

Quantitation of the primer extension products showed that mRNA and cRNA levels were considerably more reduced than was vRNA in the presence of 0.1 μg to 0.5 μg of 1/244 plasmid DNA (Fig. 5a, b). Addition of 0.1 μg of 1/244 plasmid DNA reduced mRNA and cRNA levels to 13% and 10% of the control, respectively, while the level of vRNA was reduced to only 54% of the control (vRNA:mRNA *p* = 0.0052, vRNA:cRNA *p* = 0.016). However, 0.5 μg and 1 μg of 1/244 reduced the levels of mRNA and cRNA synthesised from segment 1 by >99% while levels of vRNA remained at 14%. Thus 1/244 DI RNA has a profound effect on all RNAs synthesised from the segment 1 target, but differentially targets positive-sense RNAs. To control for the specificity of action of the inhibiting RNA, either a segment1 (encoding the intact PB2 gene) or a segment 6 (encoding the NA gene) plasmid was transfected in the place of the DI RNA plasmid. Fig. 5c-f show that segment 1 and 6 RNAs were significantly less effective at inhibiting mRNA, cRNA and vRNA expression from the segment 1 target than was 1/244 DI RNA. This is consistent with the lower level of fluorescence inhibition achieved by the segments 1 and 6 RNA (Fig. 3a). The reduction in the level of segment 1-GFP encoded mRNA and the reduction in GFP fluorescence (Figs. 3 and 5) were strongly positively correlated (R^2^ = 0.90; data not shown), confirming that fluorescence was a faithful marker of mRNA synthesis.

**Fig. 6:**
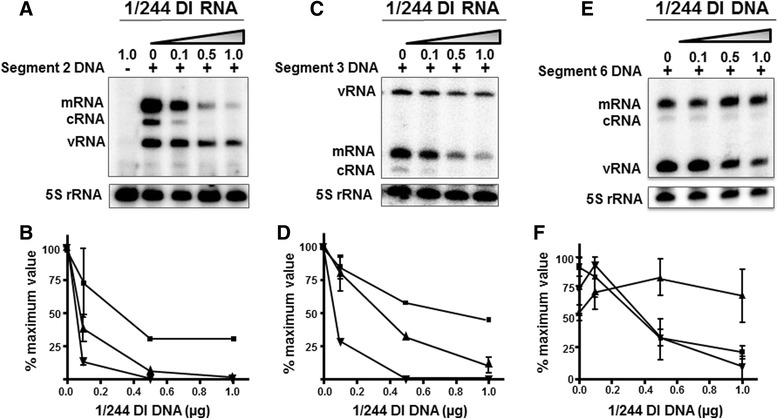
The effect of influenza 1/244 DI RNA on the level of RNA transcribed from influenza genome segments 2, 3 and 6. 293T cells were transfected with plasmids as described in Fig. 3. Analysis of RNA derived from segment 2 is shown in panel (**a**), segment 3 in panel (**c**) and segment 6 in panel (**d**) in the presence of increasing amounts of 1/244 DI RNA. Quantitation of viral RNA from two independent experiments is shown for segment 2 (**b**) and segment 3 (**d**) and three independent experiments for segment 6 (**f**). The error bars represent the range of data for three experiments. vRNA (■), mRNA (▲) and cRNA (▼)

**Fig. 7:**
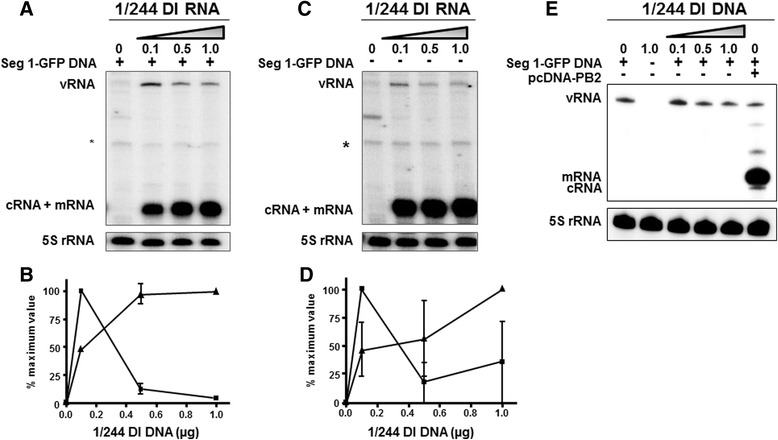
The effect of 1/244 DI RNA on its own levels of transcribed RNA in the presence or absence of seg 1-GFP. Human 293T cells were transfected with pPolI-244 and expression plasmids as described in Fig. 3. Primer extension analysis of levels of RNA transcribed from 1/244 DI RNA in the presence (**a**) or absence (**c**) of 1.0 μg segment 1-GFP. The faint band indicated by (*) is the extension product from the pcDNA PB2 expression plasmid used in the transfection. **b** shows the quantitation of viral RNA levels from four independent experiments with the standard error of the mean, and (**d**) shows the range for two replicates. vRNA (■), positive-sense RNA (cRNA + mRNA) (▲). The effect of omitting the plasmid expressing the influenza PB2 protein on virus RNA synthesis is shown in panel (**e**)

To determine if 1/244 DI RNA-mediated inhibition of RNA synthesis is segment specific, the segment 1 target (Seg1-GFP) was replaced with a plasmid directing the synthesis of either full-length segment 2, segment 3 or segment 6 vRNAs. Fig. 6a-d shows that 1/244 DI RNA reduced the levels of all three RNAs synthesized by segment 2 or segment 3, whereas segment 6 mRNA production was unaffected even at the highest amount of 1/244 DI plasmid transfected (1 μg), cRNA and vRNA levels were reduced to 12% and 23% of the control value, respectively. Although inhibition of segment 2-derived RNAs by 1/244 DI RNA (Fig. 6a, b) more closely resembled that seen for Seg1-GFP (Fig. 5a, b) than for segment 3 or 6 target RNAs (Fig. 6c-f), four-fold more 1/244 plasmid DNA was required to reduce segment 2 mRNA levels to 13% of the control than was needed for Seg 1-GFP. The trend of reduction of mRNA, cRNA and vRNA levels with the segment 3 target in duplicate samples was similar though slightly less pronounced (Fig. 7c, d). Overall, the data are consistent with a mechanism whereby 1/244 DI RNA reduces levels of mRNA synthesized from segments 1, 2 and 3, but not from segment 6.

### 1/244 DI RNA inhibits synthesis of its own negative-sense vRNA but not its own positive-sense RNA

In light of the ability of 1/244 DI RNA to differentially reduce the level of segment 1-encoded RNAs, we investigated whether the levels of positive- and negative-sense RNAs synthesised from the 1/244 DI RNA itself were also affected. RNA was extracted from 293T cells transfected with increasing levels of 1/244 DI RNA, and plasmids expressing PB1, PB2, PA and NP proteins, in the presence or absence of Seg1-GFP DNA (Fig. 7a, c). Although we found previously that we could detect vRNA, cRNA and mRNA using primer extension, in these experiments the gel electrophoresis could not distinguish the positive-sense cRNA and mRNA. However, Fig. 7a, b show that in the presence of the Seg 1-GFP target, 1/244 DI positive-sense RNA levels increased in proportion to the amount of transfected 1/244 DI plasmid. Thus these were maximal at the same time as the Seg 1-GFP mRNA and cRNA levels were at a minimum as shown in Fig. 5a, b. This shows that 1/244 DI RNA does not inhibit all influenza polymerase-directed transcription. However, the level of 1/244 DI-specific vRNA was reproducibly maximal with 0.1 μg 1/244 plasmid DNA, but decreased to 12% of the maximum value with 0.5 μg plasmid (vRNA: positive-sense RNA *p* = 0.0002), and to 4% with 1 μg plasmid (Fig. 7b) demonstrating that a high concentration of 1/244 DI RNA reduces the level of its own de novo produced vRNA. When the 1/244 plasmid DNA was titrated in cells in the absence of any target RNA the resulting levels of 1/244 positive-sense RNA and vRNA were similar to those in the presence of segment 1 target RNA (Fig. 7c, d). The effects of 1/244 DI RNA on RNA synthesis were entirely dependent on transcription of the target RNA carried out by the virus polymerase as omission of the expression plasmid that provides the PB2 protein that forms part of the virus RNA polymerase, and which is encoded by segment 1, resulted in no expression of Seg1-GFP mRNA or cRNA, although low levels of vRNA transcribed directly from the input plasmid DNA were detected as expected (Fig. 7e).

## Discussion

Despite many years of investigation, the mechanisms of the interfering action of DI influenza viruses in vitro and protection from disease in vivo remain elusive [58–62]. It is commonly held that the small size of the DI RNA allows it to outcompete the full-length genome, and that the proportion of virus particles containing DI genomes simply reflects the relative levels of DI and intact genomes present within infected cells [63]. A second hypothesis is that DI RNA has a comparative advantage over full-length genomes for a limiting viral factor (such as the NP protein) or a host factor [64]. However, there is little experimental evidence to support either of these hypotheses in regard to DI genomes in general, or to influenza DI genomes in particular. A more recent third hypothesis suggests that the influenza DI RNA interferes at the level of packaging of genomic RNAs into virions [57]. There have also been suggestions that different influenza DI sequences have different biological properties [41]. Here we investigated the mechanism of action of the cloned influenza A segment 1-derived 1/244 DI virus and demonstrated that its DI RNA inhibits infectious influenza A viruses at the level of packaging of virion RNA and by specific inhibition of virus RNA synthesis.

We have demonstrated that the activity of 1/244 DI is solely an RNA-based phenomenon. DI mRNAs that retain the AUG initiation codon of the major open reading frame have the potential to be translated into truncated peptides, as already demonstrated for some other segment 1-derived DI RNAs [65], and analogous short polypeptides containing the PA protein binding domain of the PB1 protein strongly inhibited influenza A RNA polymerase activity [66, 67]. A truncated form of the PB2 protein was also shown to be a potent inducer of interferon that could inhibit influenza A replication [68]. Mutation of the AUG initiation codon for PB2 and two further downstream in-frame AUG codons that could direct synthesis of short polypeptides from the PB2 ORF in the 1/244 DI RNA segment (Fig. 1b) generated a 1/244 AUG knock-out DI RNA that was replicated and packaged and indistinguishable in action from that of the parental 1/244 DI RNA. Virus containing the 1/244 AUG KO DI RNA protected mice from an influenza challenge to a similar extent to 1/244 DI virus. DI 1/244 KO RNA synthesised vRNA and mRNA to similar levels, and inhibited GFP expression from segment 1 to a similar extent as did 1/244 DI RNA.

Analysis of RNA present in virus particles suggests that 1/244 DI RNA is preferentially packaged over its cognate full-length segment 1 RNA into nascent virions (Fig. 4). Thus, in infected cells 1/244 DI RNA is found as a virion RNP and acts in a segment-specific manner similar to that reported for the segment 1-derived 317 DI RNA in a non-cloned virus population [57], and for cloned 317 DI virus [69]. This is consistent with current models suggesting that influenza genome segments form arrays consisting of a single copy of each segment (whether full-length or DI) and that these arrays act as single structures which become packaged into new virus particles [70–73]. These data indicate that competition for packaging with the cognate full-length genomic RNA is likely to be a common feature of all influenza DI RNAs.

It was not possible to use in vitro infection to study interference by the 1/244 DI genome due to the natural occurrence of DI RNAs in all infectious influenza preparations, their activity masking any signal from the DI RNA synthesized from the transfected plasmid. We therefore used the well-characterised RNP reconstitution assay to investigate viral RNA synthesis in the absence of virion production. While initial transcription events in this system require PolI driven plasmid transcription, for most of the 48 h time course of the assay virus proteins are synthesised from the newly synthesised virus RNAs as they are in a natural infection. Analysis of the effect of the 1/244 DI genome on mRNA synthesis from a segment 1 target genome RNA, measured directly or by monitoring expression of a reporter gene, showed that 1/244 DI RNA interfered with the synthesis of segment 1 (Fig. 3). The considerably weaker level of inhibition mediated by full-length segments 1, 4 and 6 confirmed that this effect was 1/244 DI RNA-specific. Inhibition seen with increasing levels of plasmid DNA expressing genome segments 1, 4 and 6 may be due to high levels of these RNAs competing for a limiting factor such as the virus polymerase complex.

The synthesis of positive- (mRNA and cRNA) and negative-sense (vRNA) RNAs are distinct processes [20, 21]. Data presented here show that 1/244 DI RNA produces positive- and negative-sense RNAs with the predicted sizes and differentially affects the steady state levels of the RNA products expressed by various targets. Increasing amounts of transfected 1/244 DI plasmid dramatically reduced full-length segment 1-derived positive-sense mRNA and cRNA levels but four-fold more plasmid DNA was required to reduce negative-sense vRNA to similar levels (Fig. 5a, b). This contrasts with an earlier report that the segment 1-derived 317 DI virus did not inhibit RNA synthesis, though in that study the DI RNA was not molecularly cloned and has a different sequence to 1/244 [57]. Surprisingly, 1/244 DI RNA also strongly inhibited mRNA synthesis from segments 2 and 3 (Fig. 6) suggesting that genome segments 1–3 (encoding components of the virus RNA polymerase) share a common feature(s) that permits the inhibitory action of segment 1 DI RNAs. This was reciprocated as DI RNAs 2/265 and 3/262 inhibited GFP expression from the segment 1 target (Fig. 3). This is the first demonstration that an influenza DI RNA can dramatically affect gene expression from a genome segment other than that from which it arose. At levels of 1/244 DI RNA which strongly reduced the level of target mRNA from segment 1, its own positive-sense RNA levels were maximal (Fig. 7a, b). Thus, 1/244 DI RNA can transcribe from itself while suppressing synthesis from the target segment 1 RNA.

These data provide the first description of a novel mechanism for preferential production of flu DI RNA compared to RNA production from the cognate genome segment. As the amount of transfected 1/244 DNA was increased there was a proportionate decrease in the mRNA, cRNA and vRNA synthesised by the segment 1 target RNA (Fig. 5) whereas, with transfection of low levels of 1/244 DNA all three RNAs transcribed from the 1/244 DI RNA template increased (Fig. 7). However, the situation is complicated as higher levels of 1/244 DI plasmid caused a reduction in the amount of DI vRNA. This reduction is not yet understood. The observed decline in DI vRNA levels suggests there could be an imbalance in the synthesis of the three DI RNAs in which vRNA, which is templated by and therefore dependent on DI cRNA, loses out. This would appear to be a self-limiting phenomenon not previously described for DI virus systems.

The reduction of mRNA levels by 1/244 DI RNA, indicated by the expression of GFP, was not observed when full-length segments 4 or 6 were used as the target, indicating that 1/244 DI RNA does not interfere with all genome segments and appears to act selectively on the synthesis of positive-sense RNA from segments 1, 2 and 3. Others have shown that segments 1, 2 and 3 direct the synthesis of considerably lower levels of mRNA relative to vRNA compared with other genome segments, and it has been suggested that segments 1–3 mRNAs are produced by primary transcription rather than transcription from newly synthesised vRNA [74, 75]. Thus transcription from the three largest genome segments and the other segments appears to differ. Data presented here suggest that DI RNAs derived from segments 1, 2 or 3 suppress transcription from segments 1, 2 and 3 by affecting this transcription process. However, they do not have this effect on segment 6 which, in common with segments 4, 5, 7 and 8, is transcribed primarily from newly synthesised genomes. Large quantities of short RNA molecules, referred to as svRNA or leRNA, are produced during influenza infection [76, 77], and may play a role in the switch from transcription to replication [76]. If correct, this raises the possibility that a DI RNA may serve as the template for the production of svRNAs, which in turn modulate the production of the replication products vRNA, cRNA, and mRNA. The mechanism(s) by which these different synthetic processes in segments 1, 2 and 3 are affected is not known, and it will be of interest to investigate if this is common to all influenza DI RNAs or is a property only of certain specific DI RNAs. That is, the huge number of DI RNAs that can be produced during an influenza infection may vary quantitatively and/or qualitatively in the interference they exert.

The ability of influenza DI RNAs to supplant their cognate genome segment during the packaging process explains their amplification in virus preparations. However, data presented here also indicate that the widely held view that interference results solely from the ability of the DI RNA to be replicated faster than the longer, cognate full-length RNA, while possibly a contributory factor, need not always be the only mechanism involved. Rather, the 1/244 DI RNA specifically targets the synthesis of full-length genome segments 1, 2 and 3. Further DI RNAs derived from segments 2 and 3 also inhibit RNA synthesis from full-length segment 1. Inhibition primarily affects segments 1–3 as RNA synthesis directed by segment 6 is inhibited to a significantly lesser degree. Taken together, the data suggest that interference depends upon a direct association between each DI RNA and segment 1. Since all virion and DI RNAs form RNP complexes with NP protein, we suggest that specific interactions reside in the DI RNA itself through an element, which might be a sequence, a motif comprising non-contiguous sequences, or a structural motif. This may be analogous to the association of genome segments through direct RNA: RNA interactions that have been described as a component of virus packaging [72, 78–83].

## Conclusions

The data presented here suggest there may be previously unknown interactions between full-length RNAs 1, 2 or 3 and their cognate DI RNAs, possibly mediated through yet to be identified recognition elements. These interactions appear to confer complex yet highly specific effects on viral transcription, and is therefore likely to be of evolutionary significance to the survival of the virus. This would suggest that DI RNAs are not a mere accident of replication, as frequently proposed, but may have evolved as a non-heritable means of negatively regulating virus infection. These findings take us closer to understanding the evolutionary significance of DI RNAs and their interactions with infectious virus, and nearer to understanding the action of antivirals based on DI RNAs.

## Abbreviations

cRNA: Positive sense antigenome RNA
DI: Defective interfering
DIG: Digoxigenin
GFP: Green fluorescent protein
ORF: Open reading frame
PCR: Polymerase chain reaction
RNP: Ribonucleoprotein
vRNA: Negative sense virion genomic RNA
VSV: Vesicular stomatitis virus
